# Polymer interdigitated pillar electrostatic (PIPE) actuators

**DOI:** 10.1038/s41378-021-00328-0

**Published:** 2022-01-31

**Authors:** Di Ni, Ronald Heisser, Benyamin Davaji, Landon Ivy, Robert Shepherd, Amit Lal

**Affiliations:** 1 School of Electrical and Computer Engineering, Cornell University, Ithaca, NY 14853 USA; 2 Sibley School of Mechanical and Aerospace Engineering, Cornell University, Ithaca, NY 14853 USA

**Keywords:** Electrical and electronic engineering, Engineering

## Abstract

This work reports a three-dimensional polymer interdigitated pillar electrostatic actuator that can produce force densities 5–10× higher than those of biological muscles. The theory of operation, scaling, and stability is investigated using analytical and FEM models. The actuator consists of two high-density arrays of interdigitated pillars that work against a restoring force generated by an integrated flexure spring. The actuator architecture enables linear actuation with higher displacements and pull-in free actuation to prevent the in-use stiction associated with other electrostatic actuators. The pillars and springs are 3D printed together in the same structure. The pillars are coated with a gold–palladium alloy layer to form conductive electrodes. The space between the pillars is filled with liquid dielectrics for higher breakdown voltages and larger electrostatic forces due to the increase in the dielectric constant. We demonstrated a prototype actuator that produced a maximum work density of 54.6 µJ/cc and an electrical-to-mechanical energy coupling factor of 32% when actuated at 4000 V. The device was operated for more than 100,000 cycles with no degradation in displacements. The flexible polymer body was robust, allowing the actuator to operate even after high mechanical force impact, which was demonstrated by operation after drop tests. As it is scaled further, the reported actuator will enable soft and flexible muscle-like actuators that can be stacked in series and parallel to scale the resulting forces. This work paves the way for high-energy density actuators for microrobotic applications.

## Introduction

Micro-to-milli-scale robotic systems display numerous advantages in applications such as disaster recovery and high-risk environmental investigation. To accomplish these tasks, robotic systems must produce high output forces with high efficiencies. Muscle-like work densities have been a goal of actuator engineers, especially in the context of winged flight systems for humans^1^. Biological muscles can generate energy densities in the range of 8–40 mJ/cc^2^. Over the past two decades, efforts have been made in insect-scale robot research programs with the goal of achieving a work density comparable to that of muscles while also maintaining flexibility. For example, directly using muscles at the animal scale^3^ and adapting muscle tissues^4^ or single muscle cells^5^ to artificial entities have been used to power the actuation of microsystems. One of the great challenges in biohybrid systems is achieving coupling between muscle tissues and microfabricated devices with sufficient force and power transmissions. The maintenance of biological cells out of their native environment is another obstacle, as muscle functionality and reliability decay ex vivo. Artificial devices replicating features of natural muscles then became a necessary replacement for microrobots. In the implementation of mm–cm scale robots, the energy density and efficiency are especially important due to the limited volume, payload, and stored energy. A completely man-made high work density, flexible, and efficient actuator can enable robotics to more closely resemble biological systems.

Although various actuators have been implemented in microsystems, actuators with a good combination of work densities and efficiencies continue to be investigated. Electromagnetic actuators are prevalent at macroscopic scales for their reliable low-voltage operation (1−100 V), but the electromagnetic force and efficiency drop dramatically as the size of the actuators scales down^6^. Piezoelectric actuators are another promising candidate due to their high-energy density. Large forces have been demonstrated in PZT bimorphs, with limited stroke distances^7,8^. To enable large displacement in a piezoelectric actuator, a long leveraging arm of a few centimeters must be integrated; this is a passive element and occupies extra space^8^. Thermally activated actuators, such as shape memory actuators^9^ and twisted artificial muscles^10^, demonstrate large forces and strains (*>*40%) but suffer from long reaction times and low efficiencies.

Electrically powered actuators provide fast actuation responses, high work densities, and high efficiencies. In addition to air-gap electrostatic actuators, actuators filled with a dielectric have been explored to increase the energy density and flexibility. Dielectric elastomer actuators (DEAs) utilize the electrostatic attraction between a hyperelastic, elastomeric dielectric insulator. DEAs use stretchable elastomers that are compliant and can undergo large strains^11^. Recent DEA designs^12^ have used hydraulically amplified soft electrostatic actuation by replacing the elastomer dielectric with a movable liquid. This actuator offers high strains (69%), lightweight construction, and damage tolerance. A limitation of DEA designs is the small electrode gap (20–300 µm) required to generate sufficient electrostatic forces. The small gaps generally limit the maximum displacements in DEAs. Another type of electrostatic actuator is the comb-drive actuator, which can achieve relatively large displacements. Schindler et al. demonstrated a MEMS gripper with a gripping force of 15 mN and a displacement of 1 mm^13^. This type of actuator is unable to be scaled in three dimensions due to the thin nature of silicon wafers and the intrinsic limitations in lithographic manufacturing processes.

In this work, we report a three-dimensional polymer interdigitated pillar electrostatic (PIPE) actuator with the potential to produce high forces and large displacements. PIPE actuators are arranged by meshing opposing arrays of polymer pillars together while avoiding contact between any two pillars. Similar to DEAs, PIPE actuators are electrostatic actuators that operate as compliant capacitors, in which two electrodes attract each other in response to external electric fields. Compared to other capacitive actuators, the interdigitated PIPE actuator design is expected to result in a 10- to 100-fold increase in the electrode surface-to-volume ratio, providing a pathway to produce forces greater than 10 N within a sub-mm^3^ actuator volume.

The use of 3D printing for fabrication is another aspect of the PIPE architecture. We use the commercially available continuous liquid interface production (CLIP) method. CLIP naturally enables the printing of 3D structures by drawing prints out of a resin bath^14^. Furthermore, CLIP has a fast printing speed and allows the use of a range of elastomer polymers^14,15^.

## Results

### Structure and operation

**Fig. 1:**
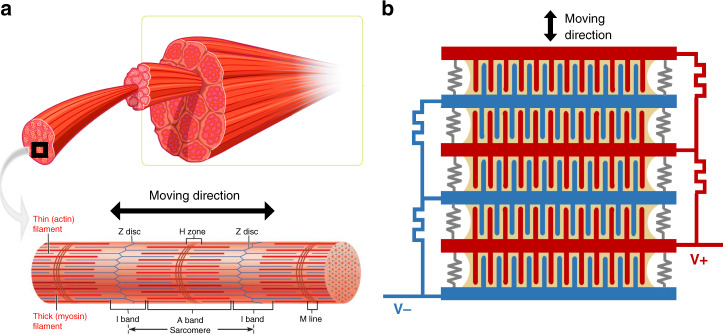
Structure of natural muscle and PIPE actuators. **a** Structure of skeletal muscles^16,17^. Each unit of muscle tissues, called a sarcomere, comprises myosin and action filaments arranged in a 3D honeycomb structure. Muscle contraction results from an interaction between actin and myosin filaments that generates movement relative to one another. **b** An array of PIPE actuators are stacked in series to scale the actuation force and displacement, which mimics the structure of natural muscles.

**Fig. 2:**
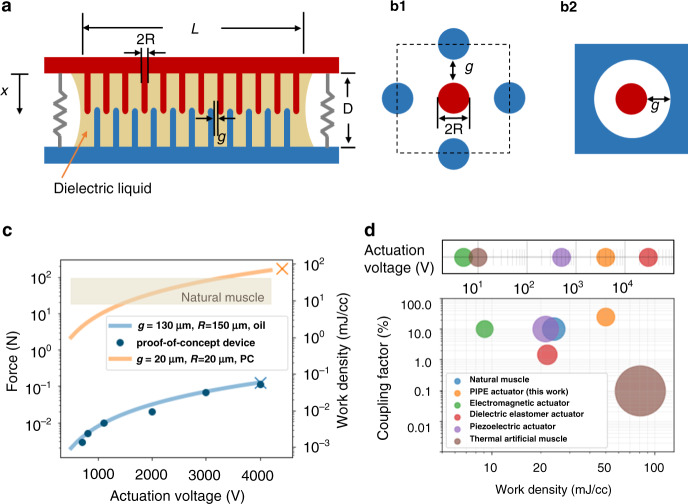
Actuator design and performance. **a** Cross-section of a PIPE actuator. A PIPE actuator consists of two electrically conductive chips with pillar arrays and an insulated spring. The spacing between two chips is filled with dielectric liquid. **b** Unit cell designs of PIPE actuators: **b1** Each pillar is surrounded by four pillars from the other chip; **b2** Each pillar is surrounded by a cylindrical cavity from the other chip. **c** Force and work density performance of two PIPE designs on a 14 × 14 mm^3^ chip. The cross sign “x” marks the breakdown points. **d** A comparison of the actuation voltage, work density, and coupling factor performance of various types of actuators and natural muscles.

The PIPE actuator design is inspired by how skeletal muscles contract. The fundamental unit of muscle, the sarcomere, consists of myosin and actin proteins intertwined in a 3D honeycomb structure^16,17^ (Fig. 1a), enabling a high surface-to-volume ratio and consequently a high-energy density. PIPE actuators mimic this 3D structure of natural muscles. The PIPE actuator (Fig. 2a) comprises two chips, each with a high density of pillars. These two chip arrays are packed, similar to myosin and actin chains. A large actuation stroke can be achieved by stacking multiple actuators (Fig. 1b) such as stacks of sarcomeres in muscle fibers.

The actuation of muscle is triggered by electrical stimuli from neurons. When muscle units are stimulated, myosin filaments slide past actin filaments. Another protein called titin functions as a molecular spring and provides passive elasticity as well as keeps myosin and actin molecules in place. Similarly, a PIPE actuator has a spring placed between the chips to connect two parts while providing the restoring force. The spring is much stiffer for motion in the lateral plane than out-of-plane to keep opposing pillars from contacting each other. When an external electric field is applied, the resulting electrostatic force between two chips pulls the movable pillar array toward the fixed pillar array without changing the pillar spacing.

Since the electrostatic force is proportional to the overlapping area of electrodes, a large surface area enables high force and work per unit of volume. Figure 2b depicts two possible unit-cell designs: (1) a pillar surrounded by four pillars from the other chip or (2) a pillar surrounded by a concentric circular enclosure. Both designs have a symmetrical geometry, which ensures that forces are balanced in the lateral plane. The pillar-to-hole design provides a larger surface area, but this paper only discusses the pillar-to-pillar design due to its manufacturing simplicity.

The space between chips is filled with a dielectric liquid to enhance electrostatic forces and to achieve higher breakdown fields. The dielectric liquid, which can be thought of as a working fluid, must simultaneously possess a large permittivity to increase the forces and a high dielectric strength to reach high electric fields. For use within an electrostatic actuator, the liquid also needs to have low conductivity to avoid electric power losses and low viscosity to reduce frictional losses. We choose dielectric oil (*ϵ*_r_ **=** 3) and propylene carbonate (*ϵ*_r_ **=** 64) as dielectric liquids^18^. Dielectric oil has been widely used in high voltage applications for decades because of its moderate permittivity and good dielectric strength, low viscosity, and very low conductivity; propylene carbonate (PC) has an ultrahigh dielectric strength and permittivity and very low viscosity. However, PC is conductive and may result in nonnegligible current losses (the characterization of the two dielectric liquids can be found in Table S1).

### Analytical model

We develop an analytical model for the parametric dependence of the force and work density of PIPE actuators. Figure 2a depicts the cross-section of a PIPE actuator, consisting of two square chips of length *L*, each with an array of *N* pillars. When no voltage is applied, the two chips are separated by a distance *D*. The pillars have a height of *h* and a radius of *R*. Opposing neighboring pillars have a gap *g*. The bottom chip acts as a mechanical ground, and the top chip is free to move. When an electrical signal is applied, the top electrode compresses the dielectrics and moves downwards by a distance of *x*.

When a voltage *V* is applied, the electrical energy stored in the actuator can be written as

1 $${{{U}}}_{\mathrm {e}} = \frac{1}{2}{{{CV}}}^2,$$

where *C* is the total capacitance. Within a cell unit (Fig. 2b1), each pillar is surrounded by four pillars from the other chip. The capacitance per unit length of a pair of pillars can be represented as^19^

2 $${{{c}}} = \gamma \frac{{\pi \epsilon _0\epsilon _{{{{\mathrm {r}}}}}}}{{\cos {{{\mathrm{h}}}}^{ - 1}\left(\frac{{{{{g}}} \,+\, 2{{{r}}}}}{{2{{{r}}}}}\right)}},$$

and *γ* is a fitting factor to be found in experiments. The total capacitance *C* of the actuator is the sum of capacitance resulting from *N* pillars:

3 $$C = Nc\left( {{{{l}}}_0 + {{{x}}}} \right)$$

where *N* is $\left( {\frac{{{{L}}}}{{2{{{g}}} \,+\, 4{{{R}}}}}} \right)^2$, *l*_0_ is the initial overlapping length between pillars, and *x* is the traveling distance. The electrostatic force acting on the actuator is

4 $$\begin{array}{*{20}{c}} {{{{F}}}_{{{{\mathrm {e}}}}} = - \frac{{{{{{\mathrm {d}}U}}}_{{{{\mathrm {e}}}}}}}{{{{{{\mathrm {d}}x}}}}} = \frac{1}{2}{{{V}}}^2\frac{{{{{{\mathrm {d}}C}}}}}{{{{{{\mathrm {d}}x}}}}} = \frac{1}{2}{{{NcV}}}^2} \end{array}$$

Substituting Eq. (2) into Eq. (4), we obtain the electrostatic force

5 $${{{F}}}_{{{\mathrm{e}}}} = \frac{1}{2}\left( {\frac{{{{L}}}}{{2{{{g}}} + 4{{{R}}}}}} \right)^2\frac{{{{{\gamma\pi }}}\epsilon _0\epsilon _{{{\mathrm{r}}}}}}{{\cos {{{\mathrm{h}}}}^{ - 1}(\frac{{{{{g}}} \,+\, 2{{{\mathrm{r}}}}}}{{2{{{r}}}}})}}{{{V}}}^2$$

This force can alternatively be expressed in terms of the electric field applied *E* = *V*/*g* as

6 $${{{F}}}_{{{\mathrm{e}}}} = \frac{1}{2}\left( {\frac{{{{L}}}}{{2 + 4{{{R}}}/{{{g}}}}}} \right)^2\frac{{{{{\gamma\pi }}}\epsilon _0\epsilon _{{{{\mathrm {r}}}}}}}{{\cos {{{\mathrm{h}}}}^{ - 1}(\frac{{{{{g}}} \,+\, 2{{{r}}}}}{{2{{{r}}}}})}}{{{E}}}^2$$

The force expressions above predict the following characteristics of PIPE actuators:

- *F*_e_ is independent of the actuation displacement *x*, enabling linear contraction.
- *F*_e_ scales linearly with the liquid dielectric constant *ϵ*_r_.
- *F*_e_ is quadratically dependent on *V*.
- Reducing the gap and radius contributes to a larger number of pillars *N*, which enables a larger surface area at a given volume.

The force expression in Eq. (6) resembles the force in DEAs, where *F*_DEA_ ***∝*** *ϵE*^2^, since both DEAs and PIPE actuators fundamentally function as compliant capacitors. However, in DEAs, the spacing between two plates is typically <0.1 mm to ensure a sufficiently strong electric field. This allows large forces and strains but small displacements. This traveling limit is not a concern in a PIPE actuator, as the top chip can move as far as the pillar height *h*. A quantitative comparison between DEAs and PIPE actuators is included in Fig. S6.

To investigate the pillar-scaling behavior, we examine the force and work density for PIPE actuators with two different pillar designs. The work density (energy per unit volume) can be calculated from ${{{w}}} = \frac{{{{{F}}}_{{{\mathrm{e}}}}{{{x}}}}}{{{{{L}}}^2{{{D}}}}}$. The two sets of pillar arrays are within a volume of 14 × 14 × 1.6 mm^3^. The pillar height is 800 µm, and the pillar radius and spacing are varied in different prints: (i) The first design serves as a prototypical device, where the pillars have a radius of 150 µm and a gap of 130 µm. This design is experimentally verified without any attempt at gap and radius optimization but is used to prove the feasibility of the high surface-to-volume ratio principle. (ii) The second design is a proposed device where the pillars have a radius of 20 µm and a gap of 20 µm. This design can be 3D printed with the two-photon polymerization technique^20^ or be fabricated with photolithography^21^. Figure 2c plots the theoretical predictions of force and work density for both designs, along with the experimental validation of the first design. A traveling distance of 150 µm, close to 20% of the height of the pillars, is assumed for the maximum work density evaluation. The measured force of the proof-of-concept device agrees well with the prediction obtained from Eq. (5). A matching factor *γ* of 0.7 is found in the force evaluation. At 4 kV, the prototypical PIPE actuator demonstrates a force of 112 mN and a work density of 54.6 µJ/cc.

Based on the validated model, the second design provides a pathway for the generation of Newtons of output force and over 40 mJ/cc work density in devices with denser pillar arrays. The yellow line in Fig. 2c shows the theoretical performance of a PIPE actuator with a pillar gap of 20 μm and a pillar radius of 20 μm. Under the same triggering voltage of 4 kV, this actuator is predicted to generate 153 N force and 73.4 mJ/cc work density using propylene carbonate as the liquid dielectric; this is a higher work density than that of natural muscles.

We compare the efficiency and work density relations of several typical actuation mechanisms in Fig. 2d^10,11,22–28^. The PIPE actuator here has a 20 μm pillar radius and a 20 μm pillar gap. Compared to other artificial actuators, the PIPE actuator promises a high work density while maintaining reasonable electrical-to-mechanical coupling.

### Prototype device demonstration

**Fig. 3:**
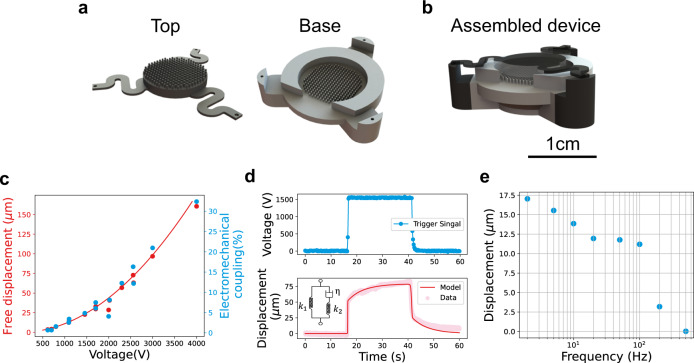
Quasi-static and dynamic characterization of PIPE actuators. **a** The top and base chips of a prototypical device manufactured with 3D printing. The top chip has an insulated spring integrated with the actuation pillar array. **b** Schematic of the assembled device. **c** The measurements of free displacement (red dots) and the electromechanical coupling efficiency (blue dots) as a function of applied voltage. The solid line shows the theoretical prediction of free displacements. **d** Actuation performance under a 1.5 kV pulse voltage signal. The resulting contraction was measured and compared with a standard linear solid mechanical model (inset). **e** The frequency responses of a PIPE actuator. The actuator was driven with square waves of 1000 V ranging from 2 to 500 Hz.

The experimental validation was performed with proof-of-concept devices fabricated using 3D continuous liquid interface processing technology (Carbon DLS), which allowed us to quickly refine the prototypes of the integrated pillars, springs, and liquid reservoirs. The prototype actuator had a circular profile (Fig. 3a) filled with 245 pillars with height of 800 µm, radius of 150 µm, and spacing of 130 µm, with an outer wall of the liquid reservoir with an exterior diameter of 2 cm and a support plate that resulted in a total height of 1.5 cm (Fig. 3b). We integrated the actuating structure and restoring spring in one monolithic design, reducing the assembly complexity. The spring consisted of three S-shaped flexures integrated on the top chip to elastically store electric energy. This S-shaped design served to move freely in the *z*-direction while keeping opposing pillars from contacting each other. The testing setup and spring characterization can be found in the Supplementary information.

The quasistatic performance of the actuator was determined by monitoring the actuation displacements with voltage ranges from 500 to 4000 V. We pulsed the input signal (~5 s) to ensure consistent actuation and electrostatic forces. Dielectric oil was used to enhance force generation. Figure 3c shows the free displacement responses after voltages were applied for 2 s. The actuation displacements were recorded using an optical displacement sensor (Model Number: MTI DTS-025-02). A maximum displacement of 160 µm was obtained at 4 kV, above which the electric field went beyond the dielectric strength of dielectric oil, and breakdown occurred. The measured displacements were used to estimate the coupling coefficient from the electrical domain (*U*_e_ **=** 1/2*CV*^2^**)** to the mechanical domain (*U*_m_ **=** *F*_e_*x*), giving a maximum electromechanical $\left({{{k}}}^2 = \frac{{{{{U}}}_{{{\mathrm{m}}}}}}{{{{{U}}}_{{{\mathrm{E}}}}}}\right)$ coupling of 32% at 4 kV.

To investigate the dynamic performance of PIPE actuators, we examined the response speed under electrical stimuli. Figure 3d plots the transient response of a PIPE actuator triggered with a 1.5 kV pulse signal. The actuator experienced a rapid contraction of 50.8 µm in the first 0.256 s, followed by another 31.8 µm increment in the following 24 s. A standard linear solid model (Fig. 3d inset) was developed to explain this nonlinear behavior: A spring *k*_1_ represents the stiffness of the folded S-shaped spring and is connected in parallel with a spring *k*_2_ and a dashpot *η* to represent the viscoelasticity caused by the UMA material (the model is discussed in detail in Supplementary information). We found good agreement between the model and experimental data, which confirms the importance of reducing the viscoelasticity of the spring materials.

The frequency responses of the PIPE actuator are shown in Fig. 3e. Driving voltages of 1 kV were applied with frequencies varying from 2 to 500 Hz. Actuation displacements were recorded at a sampling rate of 1000 Hz. The amplitudes of the displacement decreased slowly as the frequency increased up to 100 Hz, above which displacements dropped off quickly.

**Fig. 4:**
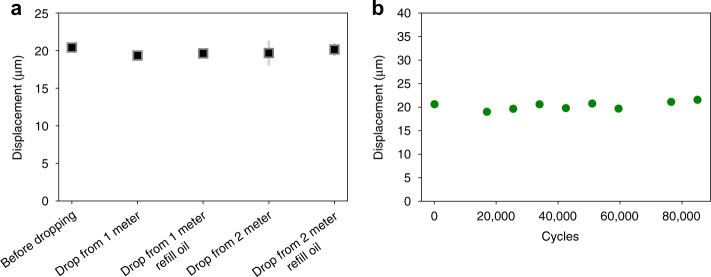
Robustness tests. **a** A PIPE actuator was dropped from 1 and 2 m heights. Actuation displacement before and after drop tests were compared. The tests for each datapoint were performed three times. **b** Cycle life of a PIPE actuator driven with 1.5 kV at 0.5 Hz.

To verify the robustness of the actuator, we performed free drop tests from heights of 1 and 2 m (Fig. 4a). The actuator was tested (i) after dropping, where the dielectric liquid might leak out due to inertial forces, and (ii) after dropping and refilling with oil. <5% displacement degradation was found after dropping, proving that the 3D printed polymer body allowed the actuator to operate even after high mechanical force impact. It also demonstrated that the interdigitated structure was able to keep most of the dielectric liquid in place because of capillary forces. The drop test indicates that a packaging method would be desirable to seal the dielectric liquid.

A cyclic test was performed for over 100,000 cycles to test the lifetime of the PIPE actuator. A 1.5 kV square wave at 0.5 Hz was applied to drive the actuator. The displacement of actuation was recorded at a 100 Hz sampling rate. Figure 4b shows negligible degradation in the displacement, which indicated that the actuator had a very long lifetime. The experiment was stopped due to laboratory equipment availability and not due to actuator failure.

## Discussion

We introduced a new class of electrostatic actuators called PIPE actuators with pillar array structures. With a great increase in the surface-to-volume ratio, this type of actuator can generate high forces and displacements in a unit of volume. The prototypical device validated the analytical model for PIPE actuators, thus promising the possibility of an actuator with a work density comparable to that of natural muscles.

The performance of the actuator also depends on the choice of spring material. As shown in the pulse test, the viscoelasticity of UMA resulted in unrecoverable deformation. We integrated the spring with the actuation pillar arrays in one print to simplify the manufacturing process. However, the spring can be manufactured separately with greater freedom in design and material selection. One idea is to use magnets as springs. With carefully defined spacing between the two magnets, they can function as a spring with negligible losses.

As a capacitive actuator, the electrostatic force and the operation voltage are closely related to the dielectric media. We used dielectric oils due to their high performance in dielectric strength, electrical isolation, and permittivity. We found that to enable better performance, propylene carbonate serves as a great candidate because of its ultrahigh dielectric strength and high permittivity. A significant drawback is that the conductivity of propylene carbonate is significant (Fig. S1). An insulating layer such as a parylene-C coating can be deposited to reduce current losses. However, adding an insulator layer reduces the voltage across the liquid dielectric.

In this paper, we achieved a maximum work density of 54.6 µJ/cc, which is still smaller than the muscle work density. However, we provided a pathway to much higher densities. In the future, the gaps between the pillars and the pillar diameter can be reduced by using higher resolution 3D printing. For example, using 3D printers utilizing two-photon writing can enable very small diameter structures, in the range of a few hundred nanometers^20^. Similarly, using DRIE etching, we can form very high-density pillar structures^29^. The challenge here is the alignment of the top and base chips. Apart from the alignment tricks to be introduced in the “Methods” section, tools such as a flip-chip bonder can be used to precisely move and bond the two chips with micrometer precision.

## Material and methods

**Fig. 5:**
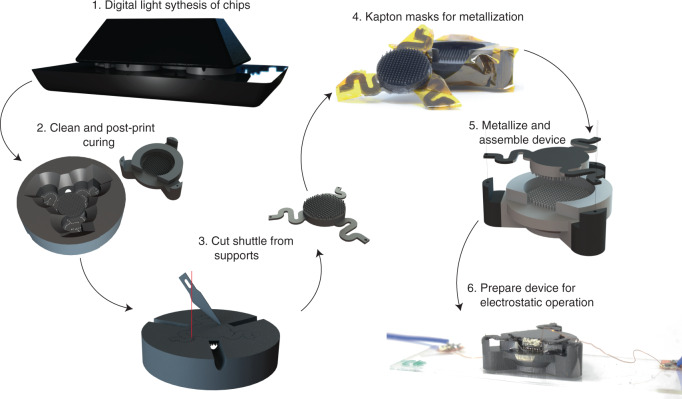
Fabrication process for PIPE actuators. (1 and 2) Actuators were printed using stereolithography, following by UV cure. (3) The top chip was released from the protective surroundings. (4) Kapton tape was applied as masks and enabled selective metal deposition. (5) A layer of AuPd (alloy) was sputtered on actuators. (6) Two components were aligned and bonded. Magnetic wires were attached to the top and bottom chip using sliver epoxy.

Fabrication (Fig. 5) began with carbon 3D liquid urethane methacrylate (UMA) resin printing (step 1), in which, with the Carbon M1 system, we printed ~5 full devices (10 parts) in under 15 min. Because thin UMA geometries tended to warp during postprint curing, the base of each chip was designed to be thick (3 mm) to ensure reasonable pillar parallelism. We printed arrays containing the S-spring with an additional stabilizing ring to protect the springs from unwanted warping while the part was in its “green state” (step 2). The excess resin was removed from the printed components using an IPA orbital wash and additional targeted IPA jetting with a Waterpik™. We then blow-dried and further UV cured the components for ~3 min. Stabilizing rings were removed using laser cutting or hand cutting with a hobby knife (step 3). For selective metal electrode deposition, we applied polyimide tape (Kapton) to the springs and mating posts to maintain electrical isolation between the top and bottom pillars (step 4). After degassing the components in a vacuum oven, we sputter-deposited Au/Pd alloy on the unmated pillar arrays for 30 min (Hummer Au/Pd Sputtering System), achieving a conformal coating with a conductivity of 10^−4^ S/m on the 3D printed UMA surface (step 5). A simple “tape test” confirmed reliable coating adhesion to the 3D printed parts. After removing the masking tape, we glued magnetic wires to the chips using silver epoxy (MGChemicals). Each half of the PIPE actuator was then ready to be aligned and bonded (step 6).

The alignment process consisted of two steps: (i) The top component was placed onto the bottom component under the guidance of optical alignment holes. (ii) The top chip was then more finely aligned under electrical impedance measurement. We monitored the capacitance and conductivity between the two chips with a multifrequency LCR meter (HP 4275A). At a given spacing between pillars, the capacitance was maximized when the pillars were well aligned. After fine alignment, methacrylate glue was used to bond the top chip to mounting posts.

## Supplementary information

Graphical abstract

SupplementaryInformation_revised - Marked Up

SupplementaryInformation_revised
