# Supplementary figures and images for: Polymer interdigitated pillar electrostatic (PIPE) actuators
Source: Microsyst Nanoeng. 2022 Jan 31;8:18. doi: 10.1038/s41378-021-00328-0 (PMC8801513; doi:10.1038/s41378-021-00328-0)

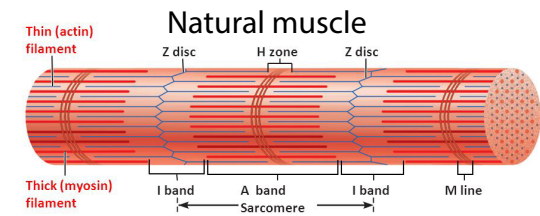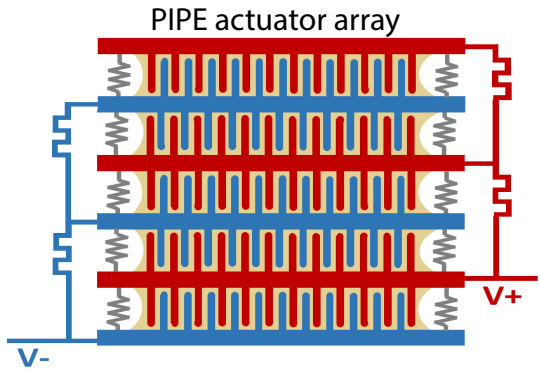

### 3D printed PIPE actuator

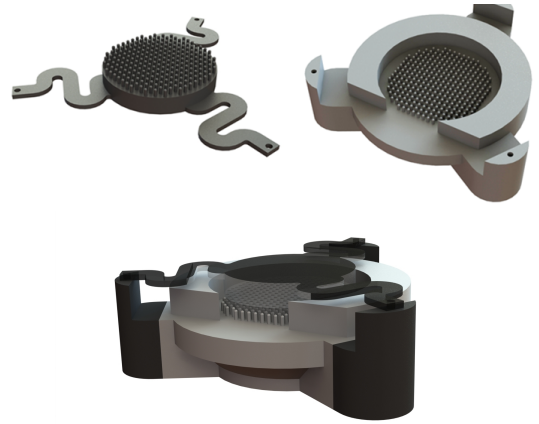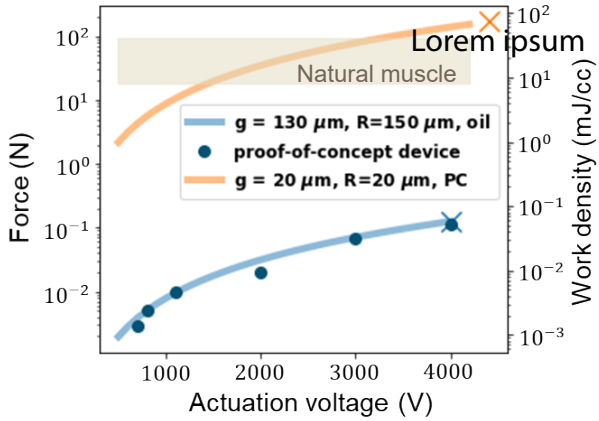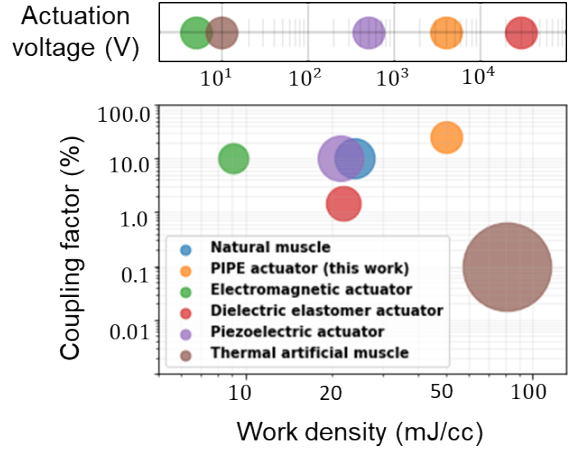

Supplement: Supplementary file 1 — Graphical abstract [file 41378_2021_328_MOESM1_ESM.pdf]
