# Supplementary material for: Polymer interdigitated pillar electrostatic (PIPE) actuators
Source: Microsyst Nanoeng. 2022 Jan 31;8:18. doi: 10.1038/s41378-021-00328-0 (PMC8801513; doi:10.1038/s41378-021-00328-0)
Supplement: Supplementary file 3 — SupplementaryInformation_revised [file 41378_2021_328_MOESM3_ESM.docx]

**Supplementary Information for**

**Polymer Interdigitated Pillar Electrostatic (PIPE) Actuators**

Di Ni1, Ronald Heisser2, Benyamin Davaji1, Landon Ivy1, Robert Shepherd2 & Amit Lal1

1*School of Electrical and Computer Engineering, Cornell University, Ithaca, NY, United States* 2*Sibley School of Mechanical and Aerospace Engineering, Cornell University, Ithaca, NY, United States*

1. **Dielectric Characterization**

Dielectric liquids provide an additional opportunity to increase the work density of PIPEs as force scales linearly with the dielectric constant. In this work, we focused on two liquid dielectrics: (i) vegetable oil: oil has been widely used in high voltage applications for decades because of its moderate permittivity and good dielectric strength, low viscosity, very low conductivity, and great chemical stability; (ii) propylene carbonate (PC): PC has an ultra-high dielectric strength and permittivity, and very low viscosity. However, PC is electrically conductive and can result in conductive current losses. We characterized the permittivity, dielectric strength, and their impact on actuator performance. The actuation results with the dielectric liquids were compared to operation in the air serving as a benchmark.

Table S1. shows the permittivity and dielectric strength of the three types of dielectrics. The permittivity of the liquid was characterized by measuring the capacitance of a device before and after filling with liquid. The measured permittivity was found to be 8.7% (vegetable oil) and 45.7% (PC) smaller than the values reported^1^. These smaller values were caused by the reduction of capacitance due to the presence of parasitic air capacitors, induced from the spacing between chips where liquid was not filled completely. Another key property is the liquid’s dielectric strength. We measured the dielectric strength of air and oil, and found a good fit between measurements and reported values. Propylene carbonate was reported to have a very high dielectric strength^2^. However, it has an electrical conductivity of 10^-7^ S/m, which prevented us from testing its dielectric strength directly due to the large leakage current.

**Table S1:** Characterization of Dielectric Liquids

| Dielectrics | Permittivity from literature^1^ | Measured Permittivity | Dielectric strength [MV/m] | Measured Breakdown field [MV/m] |
| --- | --- | --- | --- | --- |
| Air | 1 | 1 | 3 | 6.32 |
| Vegetable Oil | 3.1 | 2.83 | 30 | 31 |
| Propylene Carbonate | 60 | 32.6 | 220 | - |

Figure S1 reports the amplification of the free displacement using the three types of dielectrics. An 800 Volts DC signal was applied to the actuator. Compared to operating in air, PIPE actuators demonstrated 1.34 times higher actuation in vegetable oil and 19.31 times higher actuation in propylene carbonate. However, as shown in the right-side y-axis of Fig. S1, using propylene carbonate also resulted in a non-negligible leakage current of ~ 2mA.

**Fig. S1: Dielectric liquid characterization.** Displacement amplifications due to dielectric liquids within the PIPE gap, and the associated leakage currents. A voltage of 800V was applied, and the leakage current was obtained by monitoring the voltage across a 2.5 k$\Omega$ resistor connected in series with the actuator.


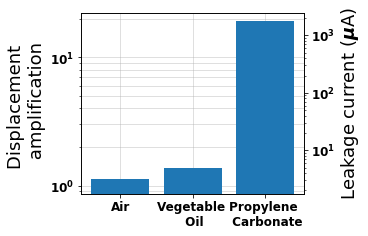


# **Experimental Testing Setup**

We tested the voltage response of PIPE actuators using the experimental setup shown in Fig. S2. The quasi-static characterization was performed by applying voltages directly from a high voltage power supply (SR PS350) (Fig. S2a). The dynamic characterization was performed (Fig. S2b) by supplying AC signals from a wave generator (Agilent 33500B), where signals were fed into a portable HV DC-to-DC converter (EMCO AG60). The high voltage converter was able to boost a low voltage of 0 - 5 volts to a high voltage of a few kilovolts. Both electrical and optical measurements were performed to characterize the actuation performances. Output voltages from the converter were measured with a high voltage oscilloscope probe (CT4025). Leakage currents through the actuator were monitored by measuring the voltage across the resistor connected in series with the actuator. Actuator displacement was measured using a laser displacement sensor (MTI DTS-025-02).


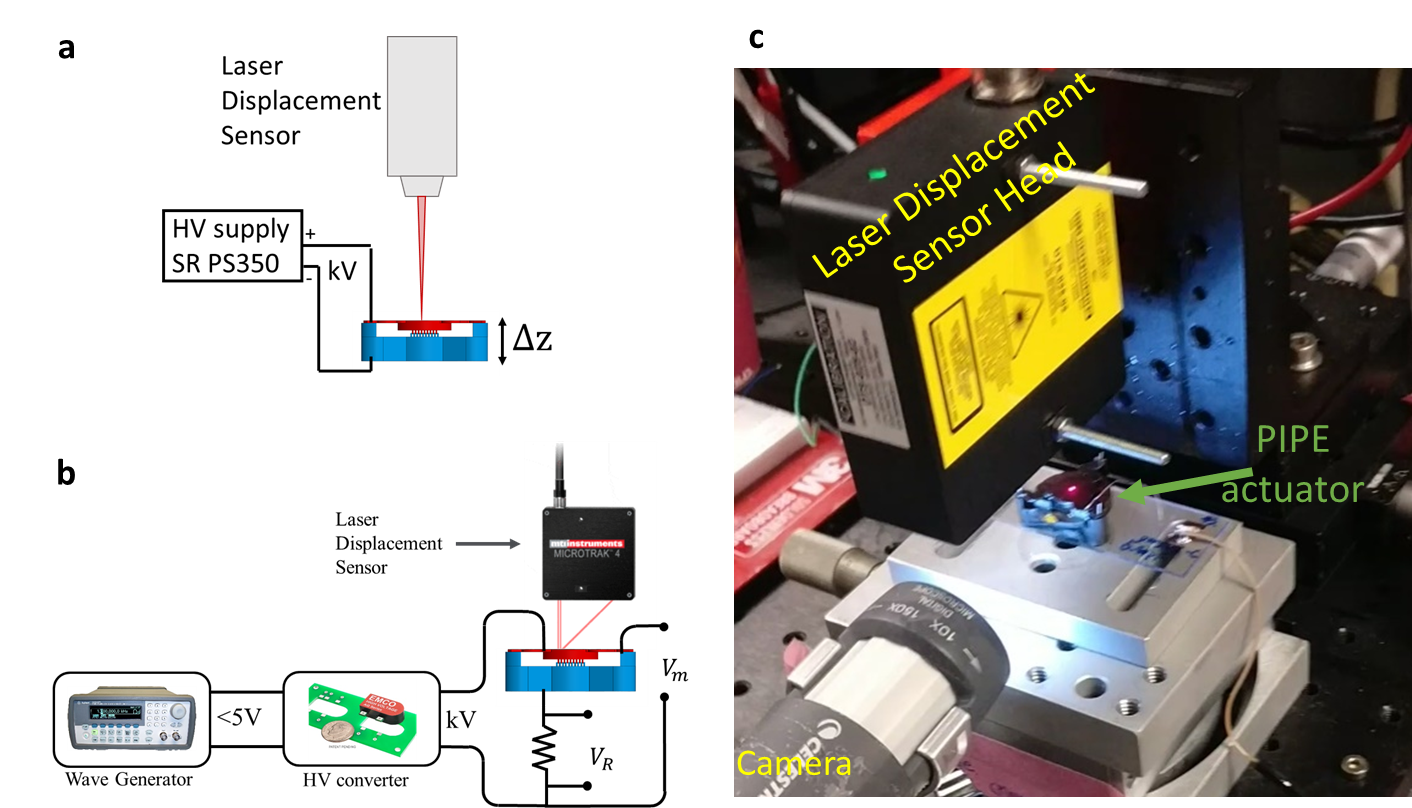


**Fig. S2: Experimental setup. a** Quasi-static characterization setup. **b** Dynamic characterization setup. **c** Picture of the optical measurement setup.

**.**

# **Spring Constant Measurement**

The spring constant of the PIPE actuator was obtained from applied mechanical load tests, assuming the spring has a linear elasticity and follows Hooke’s law:

$$F=kx$$

The force was applied by adding washers (single unit weight: 150mg) on top of the actuator. The induced displacement was recorded using the laser displacement sensor (Fig. S3a). Figure S3b shows the spring constant calculated as a function of the number of washer weights. An average spring constant of 698.5 N/m was found for the device tested in Fig. 2.


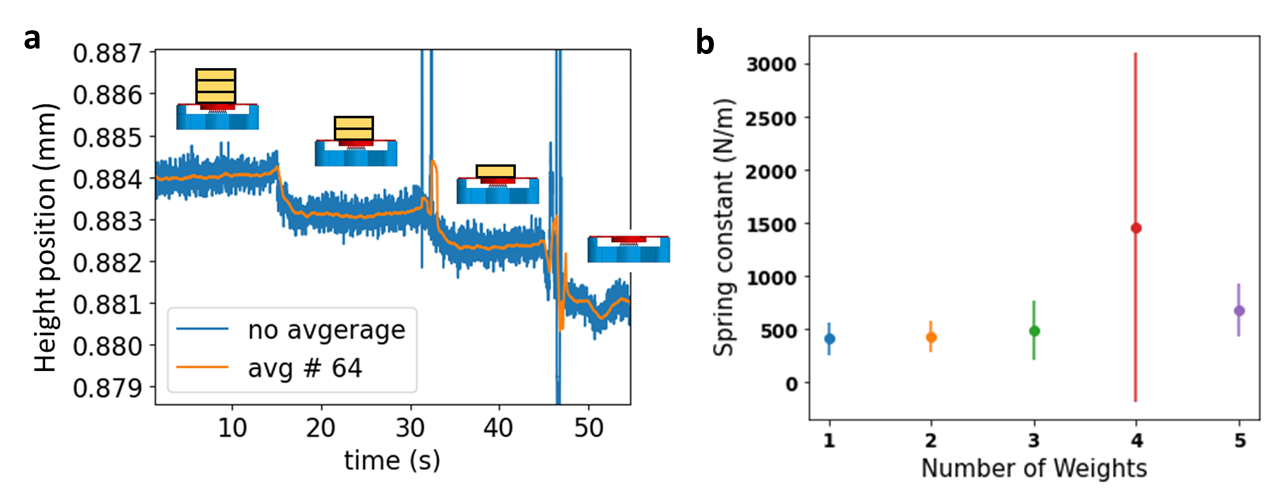


**Fig. S3: Spring constant characterization**. **a** Position changes of top chip as washer weights were added. **b** Calculated spring constant. The load tests were performed for four times.

# **FEM simulation of the lateral movements**

If the top and bottom pillar arrays are misaligned, the electrostatic forces can result in lateral bending and unwanted contact of pillars, potentially amplified due to the electrostatic pull-in instability. While we have presented the analytical model of force analysis for the well-aligned case. The pull-in stability from misalignment requires numerical study due to the complexity of having multiple pillars in the system.

We used a COMSOL model to consider this practical constraint. Figure S4a shows the architecture used in the simulation. We only considered the structure of a unit cell where a pillar is surrounded by four pillars from the other chip. The effect of the remaining pillars was not taken into consideration since the electrostatic force between two points is proportional to the inverse of the square of the distance. The top surfaces of four surrounding pillars were fixed. The bottom surface of the center pillar was fixed. The structures were merged in transformer oil, which has a dielectric strength and dielectric constant close to vegetable oil.

We considered the well-aligned case (Fig. S4b) for a PIPE actuator with pillars of 150 µm radius and 130 µm gap, and the misaligned cases (Fig. S4c) where two chips were misaligned by 50 µm and 80 µm in both x and y directions. Figure S4d shows the lateral displacement of the center pillar. When chips were well-aligned, the lateral forces were balanced out and no movement was found. A misalignment of 50 µm from the center induced a lateral pillar bending up to 3.6 µm at 10 kV, which was small compared to the gap. The pull-in instability happened when the chips were misaligned for 80 µm and actuated with voltages greater than 2 kV. The tolerance for misalignment is high for this design since a relatively large gap was used, greater care might be needed as the gap shrinks.


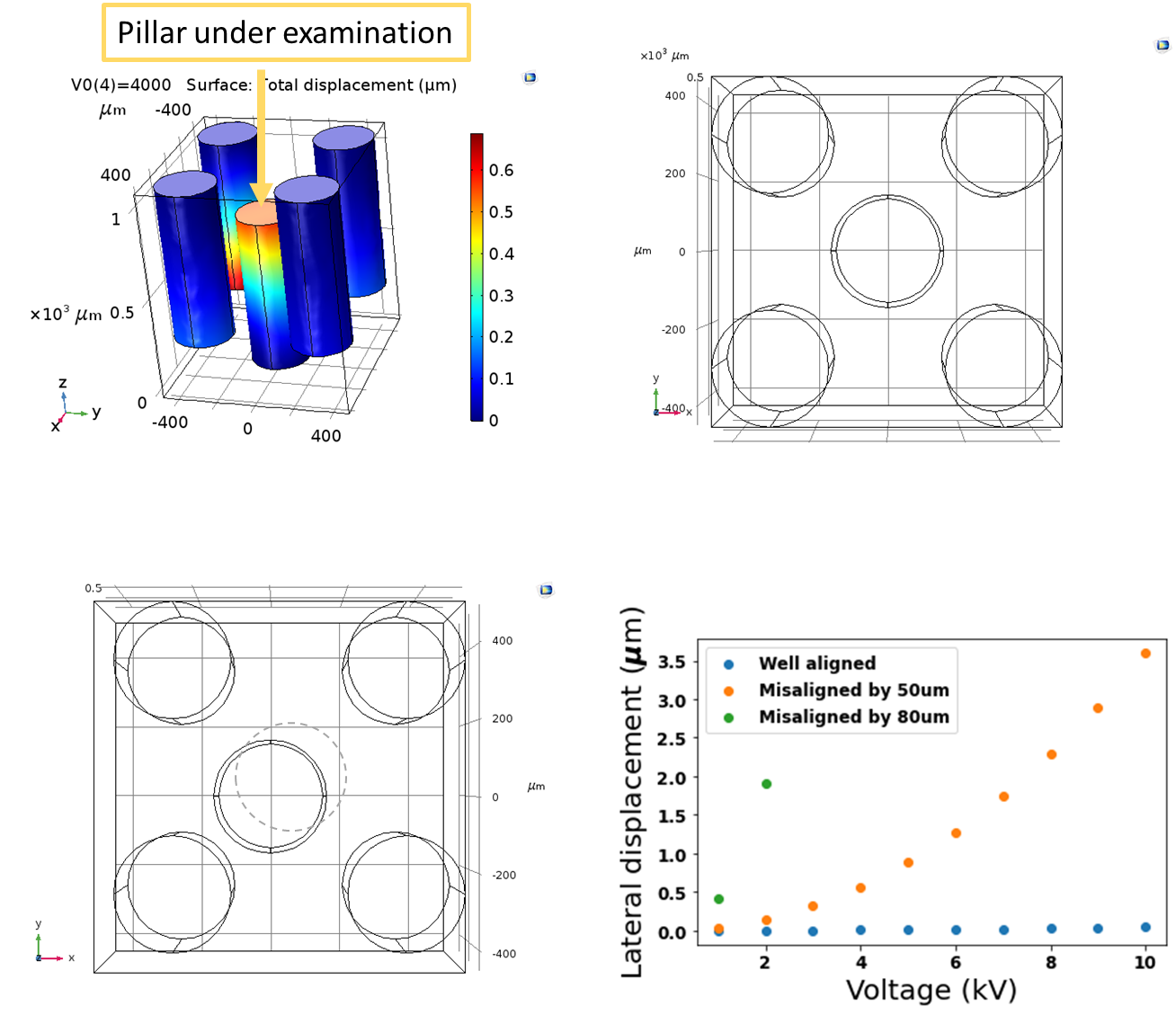


**a**

**b**

**c**

**d**

**Fig. S4 FEM simulation of lateral bending. a** COMSOL Simulation architecture. **b** Top view of well-aligned pillars. **c** Top view of a misalignment case. The pillar under examination is shifted from the center position (dashed circle). **d** COMSOL simulation of pillar’s lateral displacements.

# **Dynamic response model**

An additional practical concern in this actuator’s development is that the viscoelasticity of material may cause damping and energy loss. A time-dependent model is presented here to analyze the damped transient response of actuation. This model consists of two branches in parallel: the first branch contains a spring k1 (Effective young’s modulus $E_{1}$) representing the elasticity provided by the “S” shaped spring; the other branch contains a spring k2 (Young’s modulus$E_{2}$) and a dashpot (viscosity $\eta)$, which represents the viscoelasticity of the UMA material. The two branches have the same strain, and the total stress is the sum of stress-induced in each branch. The governing equation for the whole system can be written as^3^:

$$\sigma+\frac{\eta}{E_{2}}\dot{\sigma}=E_{1}\epsilon+\frac{\eta\left( E_{1}+E_{2} \right)}{E_{2}}\dot{\epsilon}$$

The stress $\sigma$ can be obtained assuming the electrostatic force applied is consistent. By solving the equation above, the displacement of an actuator can be found using strain $\epsilon$ caused by electrostatic force.

1. **Generalized model include liquid and material viscoelasticity**

In the model above, we ignored the liquid viscosity in the prototype device as the viscous force (~ 1 – 20 µN) was much smaller than the electrostatic force (10 – 100 mN). However, this assumption may not always hold as the gap is reduced. In this section, we will discuss the influence of viscous force and the corresponding thermal effect.

The viscous force between two planar surfaces can be estimated from:

$$F_{visoucs}=\mu A\frac{v}{g}$$

where $\mu$ is the liquid viscosity, A is the surface area of the moving chip, $v$ is the relative velocity between the two surfaces, and $g$ is the gap between pillars.

Assuming that all of the friction induced energy loss is converted into heat, raising the liquid temperature. The resulted temperature rise can be predicted from:

$$\Delta T=\frac{Q_{heat}}{mc}=\frac{F_{viscous}x}{mc}$$

Here $m$ and $c$ is the mass and thermal capacity of the liquid. Figure S5a and S5b plot the amplitudes of the viscous force and the resulting temperature rise for several gaps and velocities. The prototype device demonstrated in the paper has a pillar gap of 130 $\mu m$ and reached a maximum velocity of 200 $\mu m/s$, which corresponded to a viscous force of 14 $\mu N$ and a temperature rise of 2 $\mu℃$. These predicted changes are small enough to be ignored. However, as the gap reduces to 1 $\mu m$ and the speed increases to 2000 $\mu m/s$, the viscous force becomes comparable to the electrostatic forces.

In addition to the viscous forces in the liquid, the polymer material of the actuator also has viscous forces, in particular in the springs, which undergo time-varying strain. A generalized actuator dynamics model based on the Maxwell viscoelastic model for the polymer is shown in Fig. S5c. In addition to modeling the restoring spring and the viscoelasticity of UMA material, other losses such as the friction loss can also be considered by adding additional spring and dashpot in parallel.


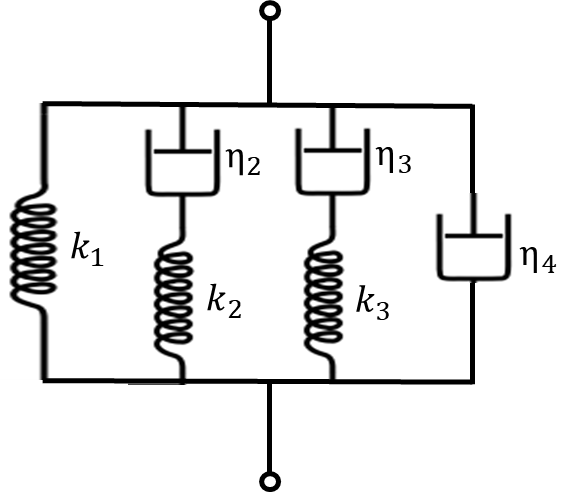


**Fig. S5: A generalized Model. a** and **b** show the viscous force and temperature rise with different pillar gap and velocity**. c** A generalized Maxwell model. $k_{1}$ represents the elasticity of the restoring spring, $k_{2}$ and $\eta_{2}$ represents the elasticity and viscous loss caused by viscoelasticity of the UMA material, $k_{3}$ and $\eta_{3}$ represents the elasticity and viscous loss caused by liquid dielectrics, $\eta_{4}$ represents all other losses.


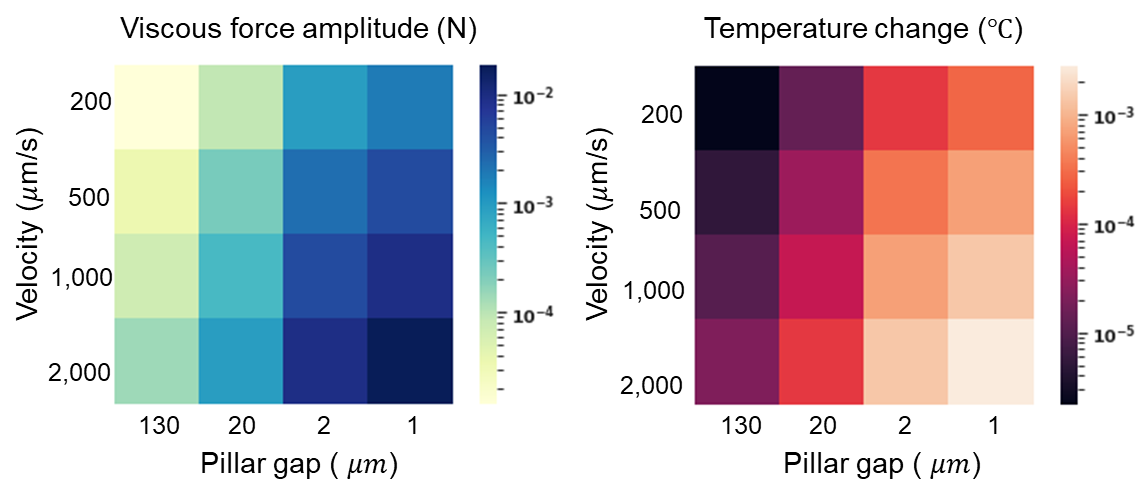


**a**

**b**

**c**

# **Detailed comparison with DEA actuators**

Figure S6 compares PIPE actuators with high strain silicone DEAs^4^ and hydraulically amplified dielectric elastomer actuators (HASELs)^5,6^. The lower boundary of the PIPE actuator uses pillar arrays with 150 µm radius, 130 µm gap, and vegetable oil as dielectrics, and the upper boundary uses pillar arrays with 50 µm radius, 50 µm gap, and propylene carbonate as dielectrics. Dielectric elastomer films with a thickness of 20$\mu m and 300 \mu m$ were used to set the boundaries of the silicone DEAs. The upper boundary of HASEL performance is set by a macro-scale actuator ( 9 cm wide, 2 cm long, 2mm thick), and the lower boundary is set by shrinking the actuator size down to 14 x 14 x 1$mm^{3}$ using the analytical model introduced in literature^7^. For a fair comparison, we assumed HASEL used propylene carbonate as liquid media, though the reported work used vegetable oil. Figure S6a shows that at 4 kV, PIPE actuators and silicone DEAs can generate dozens of Newtons of forces. HASEL has the potential to generate ~100 Newtons since a macro-scale device was used in the calculation. However, the work densities in the PIPE actuators are much larger than in the other two types of DEAs (Fig. S6b) due to the great increase of surface area in PIPE actuators, as well as the limited traveling displacement in DEAs.

**Fig. S6:** **Comparison of PIPE actuators with silicone DEAs and hydraulically amplified on:** **a** force and **b** work density.


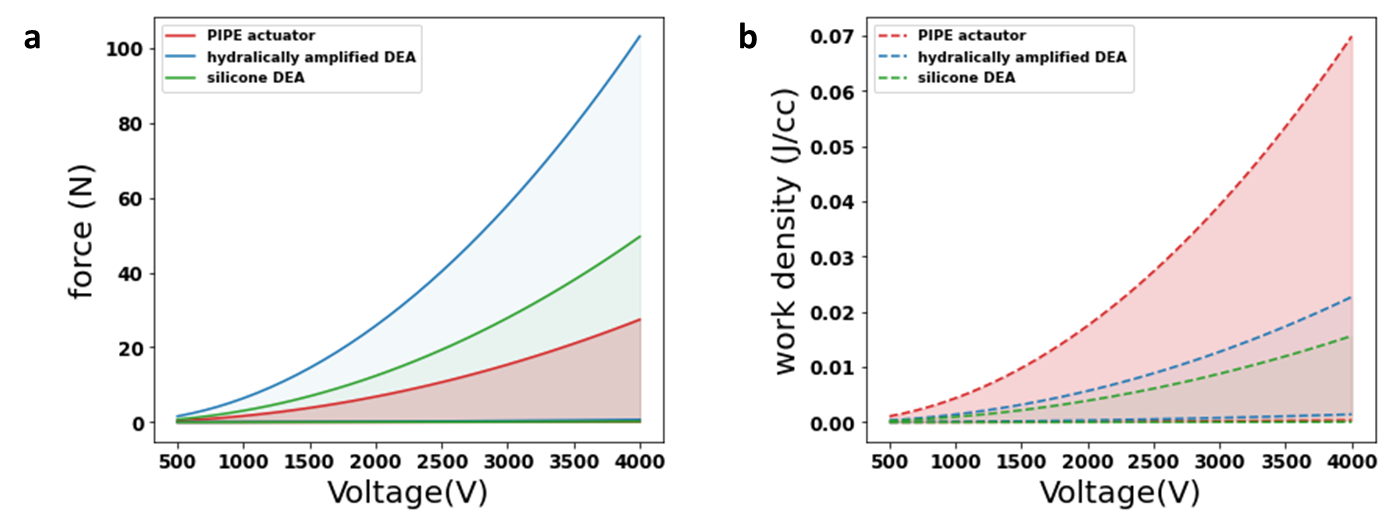


**References:**

1. Ge, B. & Ludois, D. C. Dielectric liquids for enhanced field force in macro scale direct drive electrostatic actuators and rotating machinery. *IEEE Trans. Dielectr. Electr. Insul.* **23**, 1924–1934 (2016).

2. Xiao, S. *et al.* Electrical breakdown and recovery of water and propylene carbonate. *Dig. Tech. Pap. Int. Pulsed Power Conf.* **34**, 742–745 (2007).

3. Fiugge, W. *Viscoelastici ty*. (1975). doi:10.1007/978-3-662-02276-4

4. Pelrine, R., Kornbluh, R., Pei, Q. & Joseph, J. High-speed electrically actuated elastomers with strain greater than 100%. *Science (80-. ).* **287**, 836–839 (2000).

5. Acome, E. *et al.* Hydraulically amplified self-healing electrostatic actuators with muscle-like performance. *Science (80-. ).* **359**, 61–65 (2018).

6. Kellaris, N., Venkata, V. G., Smith, G. M., Mitchell, S. K. & Keplinger, C. Peano-HASEL actuators: Muscle-mimetic, electrohydraulic transducers that linearly contract on activation. *Sci. Robot.* **3**, 1–11 (2018).

7. Kellaris, N., Venkata, V. G., Rothemund, P. & Keplinger, C. An analytical model for the design of Peano-HASEL actuators with drastically improved performance. *Extrem. Mech. Lett.* **29**, (2019).
